# Bacteriophage administration significantly reduces *Shigella* colonization and shedding by *Shigella*-challenged mice without deleterious side effects and distortions in the gut microbiota

**DOI:** 10.1080/21597081.2015.1088124

**Published:** 2015-08-28

**Authors:** Volker Mai, Maria Ukhanova, Mary K Reinhard, Manrong Li, Alexander Sulakvelidze

**Affiliations:** 1 Department of Epidemiology and Emerging Pathogens Institute; University of Florida; Gainesville, FL USA; 2 Department of Pathology; University of Florida; Gainesville, FL USA; 3 Intralytix, Inc.; Baltimore, MD USA

**Keywords:** bacteriophage, diarrhea, phage, phagebiotics, probiotics, *Shigella*, shigellosis

## Abstract

We used a mouse model to establish safety and efficacy of a bacteriophage cocktail, ShigActive™, in reducing fecal *Shigella* counts after oral challenge with a susceptible strain. Groups of inbred C57BL/6J mice challenged with *Shigella sonnei* strain S43-NalAcR were treated with a phage cocktail (ShigActive™) composed of 5 lytic *Shigella* bacteriophages and ampicillin. The treatments were administered (i) 1 h after, (ii) 3 h after, (iii) 1 h before and after, and (iv) 1 h before bacterial challenge. The treatment regimens elicited a 10- to 100-fold reduction in the CFU's of the challenge strain in fecal and cecum specimens compared to untreated control mice, (P < 0.05). ShigActive^**TM**^ treatment was at least as effective as treatment with ampicillin but had a significantly less impact on the gut microbiota. Long-term safety studies did not identify any side effects or distortions in overall gut microbiota associated with bacteriophage administration. *Shigella* phages may be therapeutically effective in a “classical phage therapy” approach, at least during the early stages after *Shigella* ingestion. Oral prophylactic “phagebiotic” administration of lytic bacteriophages may help to maintain a healthy gut microbiota by killing specifically targeted bacterial pathogens in the GI tract, without deleterious side effects and without altering the normal gut microbiota.

## Introduction

Bacterial diseases of the gastrointestinal (GI) tract continue to be a major worldwide cause of human morbidity and mortality. Among various enteric pathogens, *Shigella* spp. are some of the most common and deadly bacterial pathogens in the world, responsible for ca. 165 million worldwide cases annually, ca. 163 million of which occur in developing countries and causing >one million deaths.^1^ In the US, *Shigella* is estimated to cause ca 500,000 illnesses/year with more than 5,400 hospitalizations and 38 deaths.^2^ During the last several decades, considerable effort has gone into developing *Shigella* vaccines, and some promising vaccines have been identified.^3,4^ However, the lack of shared immunoprotective epitopes among *Shigella* serogroups and serotypes has presented a formidable challenge to vaccine development by limiting the broad efficacy of new vaccines, especially against strains that are antigenically distinct from those against which vaccine development efforts have been focused. Antibiotics can be used to treat shigellosis; however, resistance has emerged for the currently recommended antibiotics, such as fluoroquinolones, azithromycin, and third generation cephalosporins.^5^ For example, according to a recent report from the CDC, *Shigella sonnei* resistant to ciprofloxacin sickened 243 people in 32 states and Puerto Rico between May 2014 and February 2015.^6^ As a result, the CDC recommended decreasing the use of antibiotics to treat mild forms of shigellosis. In the same context, a World Health Organization report^7^ recently underscored that the increasing prevalence of multi-antibiotic-resistant bacteria in developing and industrialized countries threatens the availability of effective and affordable treatment, potentially increasing morbidity and mortality associated with various bacterial infections including shigellosis. Therefore, alternative approaches for reducing the incidence and severity of shigellosis are urgently needed. One possible approach is to use bacteriophages capable of killing *Shigella* in the GI tract during the early stages of shigellosis (i.e., a “classical phage therapy” approach) and/or before *Shigella* colonize the GI tract and cause disease (i.e., a prophylactic “phagebiotic” approach).

Bacteriophages are bacterial viruses that are arguably the oldest and most ubiquitous organisms on Earth.^8^ In contrast to antibiotics, lytic phages are fairly specific, usually targeting only a subgroup of strains within one bacterial species or across closely-related species. Their remarkable antibacterial activity prompted the use of “phage therapy” for treating various bacterial human diseases. While their use gradually declined in the West after antibiotics became widely available, use continued in the former Soviet Union and in several Eastern European countries.^9-12^ The first therapeutic application of bacteriophages in humans was for the treatment of shigellosis.^13^ *Shigella* phages were successfully and extensively used to treat shigellosis worldwide during the 1930s and 1940s, and their use has continued in the former Soviet Union and in some Eastern European countries.^9-11,14-18^ Military and civilian practitioners in the former Soviet Union commonly used *Shigella* phages to treat or prevent bacterial dysentery. ^19,20^ In a trial comparing 13,913 people prophylactically treated with a *Shigella* phage preparation to 12,690 controls, phage administration reduced the incidence of dysentery, most effectively in children <9 years of age (incidence of 0.3% *vs*. 5.2%).^21^ These results correlate well with those reported in other Soviet publications evaluating the efficacy of phage treatment for bacterial dysentery.^22-25^ However, recent reviews^12,18,26^ suggest that most of these studies were not conducted in a rigorous, double-blind, placebo-controlled manner, and all lacked analyses of various biochemical and physiological parameters including effects on composition of normal gut microbiota. Therefore, the primary goals of the studies presented here were to (i) evaluate the ability of an orally administered *Shigella*-specific phage preparation (ShigActive™) to reduce colonization and shedding of *Shigella* in experimentally infected mice (although mice generally do not show symptoms upon infection with *Shigella*, their gut anatomy allows for investigating a reduction in *Shigella* counts, either in fecal pellets or cecal contents), and (ii) determine the safety of short- and long-term oral administration of *Shigella* phage into mice, including impact on the normal microbiota.

## Results and Discussion

### *In vitro* lytic activity of the phage preparation

The ability of ShigActive™ to lyse *Shigella* strains *in vitro* was evaluated against a collection of 65 strains of *Shigella* spp isolated from clinical cases of shigellosis in various countries and *S. sonnei* strain 9290 obtained from the ATCC. The phage cocktail (at ca. 1 × 10^9^ PFU/mL) killed 62 (95%) of all strains in that collection. The three resistant strains were *S. flexneri*; i.e., the phage cocktail killed 35 of the 38 *S. flexneri* strains (92%), and 100% of all other *Shigella* strains examined. When tested against a small number of non-*Shigella* strains using the same Spot Test assay, the phage cocktail lysed all 5 *Salmonella* and 5 *E. coli* strains, but none of the *L. monocytogenes, L. innocua*, and *S. aureus* strains examined (for the list of non-*Shigella* strains, refer to the *Materials and Methods* section). In contrast to broad-spectrum antibiotics, phages are generally more specific to their bacterial hosts. ShigActive™ lysed a small number of strains of closely-related to *Shigella* spp. *E. coli* and *Salmonella*. Additional *in vitro* susceptibility data (particularly against normal gut microbiome bacteria) must be generated, but specificity of phages which enables their use with minimal impact on the normal gut microbiome has been reported previously by other investigators (including during human clinical trials)^27,28^ and is further substantiated by our 16S rRNA sequencing-based microbiome studies reported in this manuscript.

### Short-term efficacy studies

During preliminary short-term efficacy studies, we found that 72 h after a *Shigella* inoculum of 1.2 × 10^8^ CFU/mouse a 10:1 phage: *Shigella* concentration completely eliminated bacteria in stool samples, cecal and small intestinal contents (data not shown). Consequently, we used ca. 1 × 10^9^ PFU/mouse during the short-term efficacy studies described below. A total of 61 mice were used during those studies. To determine the most effective dosage regimen, ShigActive™ was administered at the following times before and/or after challenge with *S. sonnei* strain S43-NalAcR: (i) 1 h after (N = 20), (ii) 3 h after (N = 6), (iii) 1 h before and after (N = 10), and (iv) 1 h before (N = 5). In all but one treatment group (the 3 h post-challenge treatment group), the fecal specimens obtained from *Shigella*-challenged mice treated with ShigActive™ had statistically significant lower concentrations of the challenge strain than did those of the phage-untreated control mice (p < 0.05, N = 20). The lowest fecal *Shigella* counts 24 h after challenge were observed when mice received ShigActive™ close to challenge: 1 h before, 1 h after, and 1 h before and after challenge. For example, there were 110 CFU/pellet recovered from the mice treated with ShigActive™ one hour post-challenge vs. 1,114 CFU/pellet recovered from the phage-untreated control mice (P = < 0.0001). Double dosing regimen (i.e., 1 h before and 1 h after challenge) appeared to be the most effective among the treatment regimens examined: there were 26 CFU/pellet recovered from ShigActive™-treated mice vs. 1,114 CFU/pellet recovered from phage-untreated control mice (P =< 0.0001).

**Figure 1.**
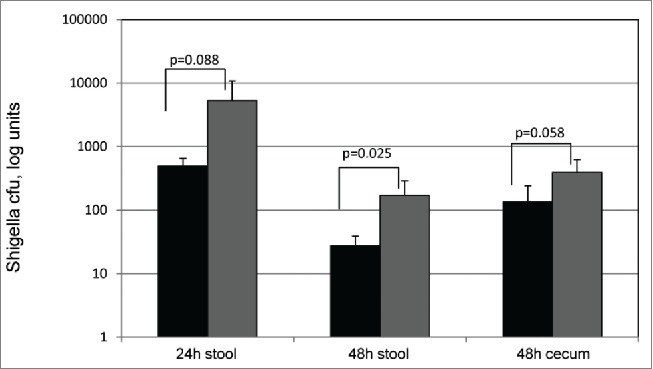
Recovery of *Shigella* strain S45 *Nal^R^* from challenged mice after treatment. Expressed as the log of CFU counts/fecal pellet or total cecum content. The results shown are 24 and 48 h post-challenge. Black: ShigActive™, Gray: Ampicillin.

When comparing the efficacy of a single administration of ShigActive™ to that of ampicillin (N = 5), ShigActive™ was more effective than ampicillin in reducing fecal *Shigella* levels in stool samples 24 h and 48 h and in cecal contents 48 h after challenge (p < 0.05). However, when both treatments were administered twice, 1 h before and 1 h after challenge, the difference in fecal *Shigella* levels was statistically significant (P = 0.025) only after 48 h (Figure 1). Ampicillin is commonly prescribed to treat *Shigella* infections; therefore, our observation that phages were at least as effective as, and possibly more effective than, ampicillin in reducing *Shigella* levels in the fecal and cecum specimens of challenged mice suggests that ShigActive™ treatment may be a viable alternative to (or be complementary to) ampicillin treatment of shigellosis especially in multi-antibiotic-resistant *Shigella* strains.

**Figure 2.**
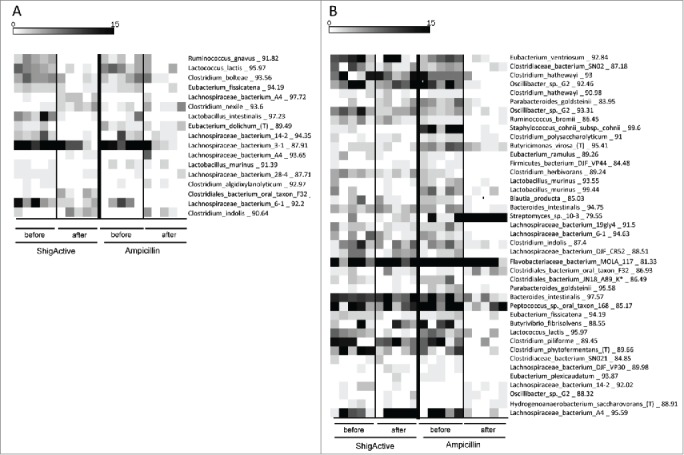
Treatment effects on OTU abundance. Heat map of selected OTUs at 95% similarity in the short term study. Each vertical column represents one sample A) OTUs that changed significantly after ShigActive administration, samples from mice receiving ampicillin are shown for comparison B) OTUs that changed significantly after ampicillin administration, samples from mice receiving ShigActive are shown for comparison. The scalebar depicts the number of sequences in each OTU detected in each sample.

Bacteriophages are much more specific then broad spectrum antibiotics; therefore, phage-treatment is commonly presumed to have less impact on the normal microbiota. We used a 16S rRNA-based sequencing approach to compare the effects of ShigActive™ treatment and ampicillin treatment on the gut microbiota of mice. We generated a total of 98566 sequences, an average of 2,464 sequences per specimen, with an average length of 435 nucleotides. Sequence reads were clustered using ESPRIT and, after removing operational taxonomic units (OTUs) containing less than 10 sequences, we retained 1,039 and 783 OTUs at the 98% and 95% similarity levels, respectively. Using the binned sequence data and the QIIME package to characterize the microbiota structure and diversity revealed that the Chao-1 diversity was significantly reduced by ampicillin (P < 0.01) but not by ShigActive™. The UniFrac distances (weighted or unweighted, both measures of β diversity) did not differ between the groups, and no clustering by treatment was detected. The predominant OTUs in both groups most closely matched to sequences from the phyla *Bacteroidetes* and *Firmicutes*. A significant increase in the proportion of *Actinobacteria* was detected only after ampicillin treatment. The number of OTU's that statistically significantly changed in prevalence after treatment was significantly higher in the ampicillin group (Figure 2). In mice receiving ampicillin we observed a 10-fold higher number of OTUs that decreased after treatment compared to phage-treated mice (366 vs. 36 at 98% similarity, and 101 vs. 40 at 95% similarity). The higher numbers of OTU's that we found to be decreased rather than increased suggest that both treatments selected *against* rather than *for* the gut bacteria. Overall, gut microbiota was less distorted in the ShigActive™-treated mice. The non-specific effect of antibiotics has been linked to dysbiosis and associated overgrowth by pathogens, invasion and translocation of toxins, and potentially life-threatening secondary infections.^29^ To the best of our knowledge, the current communication is the first experimental confirmation (on an OTU level) that treatment with *Shigella* phages has a significantly milder effect than ampicillin on the normal mammalian GI microbiota. The results are similar to those reported previously by our group for a *Listeria monocytogenes*-specific phage preparation.^30^

### Long-term toxicity studies

The long-term toxicity study was performed with a total of 38 mice. Mice receiving either PBS (N = 19) or ShigActive™ (N = 19) were sacrificed on days 7 and 28. No significant difference in body weight or any health and toxicity markers were observed between the 2 groups. There was no statistically significant difference in the body weight and weight gain of control and ShigActive™ groups at one week, and 4 weeks (P > 0.05), suggesting that bacteriophage administration did not appreciably affect energy balance. Total and differential white blood cell counts showed no statistically significant difference after 7 or 28 days between control and phage-treated groups (P > 0.05). There was no statistically significant difference in ketone content, specific gravity, pH, and protein content from urine samples of controls and cases at any time point. No abnormal levels of leukocytes, nitrites, urobilinogen, bilirubin, or glucose were detected in urine samples. There were no significant histopathological differences between the control and phage-treated groups, no pathologic lesions were identified in kidney, gastrointestinal tract, liver, spleen, heart, lung or brain. This was arguably one of the most extensive examinations of phage impact on the mammalian organism, and our data provide further evidence of the general safety of oral lytic phage treatments.^12^

During the long-term toxicity studies, we also evaluated the impact of ShigActive™ on the microbiota composition of the murine GI tract. 16S rRNA-based sequencing generated a total of 181,922 sequences, mean of 3,638 sequences per specimen, with an average length of 484 nucleotides. After removing the OTUs containing less than 10 sequences, we retained 1,139 and 753 OTUs at the 98% and 95% similarity levels, respectively. None of the diversity measures revealed significant differences between the microbiota compositions of the ShigActive™-treated normal mice and the controls. Similar observations have been made for *Escherichia coli* phages. For example, healthy adult volunteers who received T4 phage in their drinking water did not exhibit deleterious side effects or decreases in their fecal *E. coli* counts^27.^;^27^ when the same T4 phage was given to 15 healthy adults in Bangladesh, it did not alter their fecal microbiota compositions.^28^

In summary, our studies demonstrated that the ShigActive™ phage cocktail was effective in safely reducing *Shigella* counts in experimentally challenged mice. No toxic side effects of phage administration were observed during the studies, and the phage cocktail had much less impact on the normal gut microbiota than treatment with a commonly prescribed antibiotic. To the best of our knowledge, this is the first study in which the safety of short-term and long-term phage administration was comprehensively established by using a battery of microbiological, metagenomic, clinical, and biochemical tests. While the *in vivo* phage efficacy in our studies was very promising, the animal model used during our studies was an experimental ‘proof of principle’ that did not closely resemble human shigellosis (e.g., challenge with *Shigella* did not cause diarrhea or other disease symptoms in mice). Thus, the ultimate applicability of the ShigActive™ preparation for managing *Shigella* infections in humans must be established in human clinical trials. However, given the results of our studies, and an extensive (albeit mostly semi-anecdotal) scientific literature on using *Shigella* phages to prevent or treat human shigellosis, the likelihood of those future human clinical trials supporting the safety and efficacy of the approach seems excellent. If that is indeed the case, it should be possible to develop and commercialize *Shigella* phage preparations that can be used as an additional tool to help prevent shigellosis (e.g., phages administered prophylactically – i.e., “phagebiotics” approach) and/or to treat early onset of shigellosis, including cases where the etiologic agent is resistant to commonly used antibiotics (e.g., phages administered therapeutically – i.e., classical “phage therapy” approach). Such phage preparations may help reduce the significant morbidity and mortality due to *Shigella* infections – including those caused by multidrug resistant *Shigella* strains that have been emerging worldwide recently.^5-7^ The approach may also serve as the platform technology for developing a new class of prophylactic and/or therapeutic products targeting other etiologic agents that have an oral port of entry and require short-term or long-term colonization of the GI tract in order to cause disease.

## Materials and Methods

### Bacteriophage preparation

ShigActive™, the bacteriophage preparation used during our studies, is a cocktail of 5 lytic bacteriophages designated SHSML-52-1 (ATCC PTA-121241), SHFML-11 (ATCC PTA-121234), SHSML-45 (ATCC PTA-121238), SHFML-26 (ATCC PTA-121236), and SHBML-50-1 (ATCC PTA-121239). Four of the phages belong to the *Myoviridae* family and one phage (SHSML-45) belongs to the *Siphoviridae* family of double-stranded DNA phages. The phage preparation was supplied in normal saline solution (0.9% NaCl, pH 6.5-7.5), and was stored refrigerated (2–8°C) until use.

### Bacterial strains

Our *Shigella* strain collection included 64 strains of *Shigella* spp isolated from clinical cases of shigellosis in various countries (including Mali, Chile, Pakistan, Peru, Japan, and Haiti), and *S. sonnei* strain 9290 obtained from the ATCC. The strains represented all 4 *Shigella* species: *S. flexneri* (38 strains), *S. sonnei* (18 strains)*, S. dysenteriae* (5 strains), and *S. boydii* (4 strains). Our non-*Shigella* strain collection included 5 ATCC *Salmonella* strains (*S.* Typhimurium ATCC13311, *S.* Heidelberg ATCC8326, *S.* Enteritidis ATCC13067, *S.* Typhimurium ATCC6539, *S.* Hadar ATCC51956), 5 *Escherichia coli* strains (ATCC43895, ATCC35401, ATCC700728, ATCC11303, and ATCC12435), 3 *Listeria monocytogenes* strains (ATCC19117, ATCC19118*, and* ATCC19116), 2 *Listeria innocua* strains (ATCC51724 and ATCC33090), and 5 *Staphylococcus aureus* strains (ATCC25923, ATCC29213, ATCC700699, ATCC49775, ATCC14458).

Our *in vivo* challenge studies used a nalidixic acid-resistant mutant of *S. sonnei* strain S43, S43-NalAcR, which is sensitive *in vitro* to ShigActive™ in the Spot Test assay ^31^ (all 5 phages in the ShigActive™ cocktail lysed the challenge strain with approx. equal efficiency). A fresh, early log-phase culture of the S43-NalAcR strain was grown (ca. 4 h, 37°C) in LB broth containing nalidixic acid (25 ng ml^−1^), and mice were challenged *via* oral gavage with 0.9-1.3 × 10^8^ colony-forming units (CFU).

### Mouse species and housing

Inbred male C57BL/6J mice (8-weeks-old, 24.5g +/− 1.3g of weight) obtained from Jackson Laboratory (Bar Harbor, Maine) were quarantined, randomized to various cages, and allowed to acclimate to the new environment for 7 d. The mice were fed Harlan chow 7912 (Harlan Laboratories, Indianapolis, IN) *ad libitum* and provided 24 h access to fresh water. The studies were conducted at the University of Florida (UF) with a protocol following the Animal Welfare Act and the Health Research Extension Act guidelines approved by the UF's Institutional Animal Care and Use Committee. C57BL/6J mice represent an established animal model for determining microbiota effects.

### Short-term efficacy studies

*Shigella* could not be recovered 72 h after inoculation; therefore, we sacrificed mice in the short-term efficacy study after 48 h. Mice were inoculated by gavage with 0.1 ml of early log phase *S. sonnei* S43-NalAcR (ca. 0.9-1.3 × 10^8^ CFU/mouse). To identify the most effective administration regimen, ShigActive™ (ca. 1.0 × 10^9^ PFU/mouse in 0.1 ml) was administered by oral gavage before and/or after challenge as follows: (a) 1 h before, (b) 1 h after, (c) 3 h after, and (d) 1 h before and 1 h after bacterial challenge. Mice randomized to the antibiotic group received ampicillin doses (25 mg kg^−1^ in 0.1 ml) by oral gavage 1 h before and 1 h after *Shigella* challenge. Stool samples were collected before treatment, 24 and 48 h after treatment, mice were then sacrificed by CO_2_ inhalation and cervical dislocation. Blood, cecum contents, and tissue samples were collected upon sacrifice.

### Long-term toxicity studies

The duration of the long-term toxicity studies was 28 d. Starting on day 1, mice received ShigActive™ (ca. 1 × 10^9^ PFU/mouse, in 1 ml) twice a day, by oral gavage, for the first 7 d. From day 8 onwards, mice received the same dose of ShigActive™ once every other day for an additional 3 weeks. Fresh stool specimens were collected before ShigActive™ administration on days 7 and 28. On day 28, the mice were sacrificed and blood, cecum contents, and tissue specimens were collected and analyzed as described below.

### *Shigella* enumeration

Diluted aliquots (0.1 ml) of stool and cecum suspensions prepared in phosphate-buffered saline (PBS) were spread on McConkey agar supplemented with nalidixic acid (25 ng ml^−1^, to select for *S. sonnei* strain S43-NalAcR colonies), and the Petri dishes containing the inoculated medium were incubated at 37°C for ca. 36 h. The number of CFU g^−1^ of specimen was calculated after counting the number of *Shigella* colonies.

### Microbiota analyses

For the short term study, samples were collected at baseline and after 48 h in 2 independent experiments from a total of 5 control (PBS) 10 ShigActive-treated and 5 Ampicillin-treated animals. For the long-term study, samples were collected at baseline and after 28 days from 5 control (PBS) and 5 ShigActive-treated mice. DNA was extracted using a modified Qiagen stool DNA protocol.^32^ We used DGGE analysis ^33^ for initial quality control. DNA was amplified using sequencing primers 27F (AGAGTTTGATCCTGGCTCA) and 533R (TTACCGCGGCTGCTGGCAC) to which titanium adaptor sequences and barcodes were added. Purified PCR products were pooled in equimolar amounts for sequencing using 454-Titanium chemistry (Roche). The conditions and procedures for emPCR and bead enrichment were as described in the Roche protocol. Low quality sequences or sequences with lengths of <100 nucleotides were removed from the resulting raw data set. ESPRIT-tree^34^ was used to bin sequences into Operational Taxonomic Units (OTUs). We used QIIME ^35^ to calculate the Chao rarefaction diversity and UniFrac distances. ^36^

### Blood, urine, and tissue analyses

#### Hematological analyses

Blood specimens were drawn into vials containing potassium EDTA, complete blood count (CBC) profiles were determined with Hemavet 1700 (Drew Scientific, Oxford, CT), and plasma chemistry was obtained with VetAce (ALFA Wasserman, West Caudwell, NJ).

#### Urinalyses

After placing mice individually onto Glad cling wrap outside of their cages, urine specimens were aspirated (with a micropipette) into clean Eppendorf tubes and analyzed with Urine Reagent Strips (Fisherbrand, Hannover, Germany) designed to quantify leukocytes, nitrite, urobilinogen, protein, pH, blood, specific gravity, ketones, bilirubin, and glucose levels in the specimens.

#### Tissue analyses

After sacrificing the mice, specimens of their kidneys, livers, spleens, GI tracts, hearts, lungs, and brains were fixed in 10% buffered formalin for a minimum of 48 h. The fixed tissue specimens were processed according to standard procedures and stained with hematoxylin and eosin ^37^, after which they were coded and examined, by a board-certified laboratory veterinarian, for histopathological changes.

### Statistical analyses

Two-tailed t-tests were used for (i) concentrations of *S. sonnei* strain S43- NalAcR, and (ii) weight, hematological and urinalysis data. Microbiota diversity was determined using the Shannon-Weiner and Simpson (1/D) diversity indexes. Unifrac based *p*-values were used to evaluate differences in overall microbiota composition.
